# *In Vitro* Interaction of the Housekeeping SecA1 with the Accessory SecA2 Protein of *Mycobacterium tuberculosis*

**DOI:** 10.1371/journal.pone.0128788

**Published:** 2015-06-05

**Authors:** Irfan Prabudiansyah, Ilja Kusters, Arnold J. M. Driessen

**Affiliations:** Molecular Microbiology, Groningen Biomolecular Sciences and Biotechnology Institute and Zernike Institute for Advanced Materials, Nijenborgh 7, 9727 AG Groningen, The Netherlands; Centre National de la Recherche Scientifique, Aix-Marseille Université, FRANCE

## Abstract

The majority of proteins that are secreted across the bacterial cytoplasmic membrane leave the cell via the Sec pathway, which in its minimal form consists of the dimeric ATP-driven motor protein SecA that associates with the protein-conducting membrane pore SecYEG. Some Gram-positive bacteria contain two homologues of SecA, termed SecA1 and SecA2. SecA1 is the essential housekeeping protein, whereas SecA2 is not essential but is involved in the translocation of a subset of proteins, including various virulence factors. Some SecA2 containing bacteria also harbor a homologous SecY2 protein that may form a separate translocase. Interestingly, mycobacteria contain only one SecY protein and thus both SecA1 and SecA2 are required to interact with SecYEG, either individually or together as a heterodimer. In order to address whether SecA1 and SecA2 cooperate during secretion of SecA2 dependent proteins, we examined the oligomeric state of SecA1 and SecA2 of *Mycobacterium tuberculosis* and their interactions with SecA2 and the cognate SecA1, respectively. We conclude that both SecA1 and SecA2 individually form homodimers in solution but when both proteins are present simultaneously, they form dissociable heterodimers.

## Introduction

**Fig 1:**
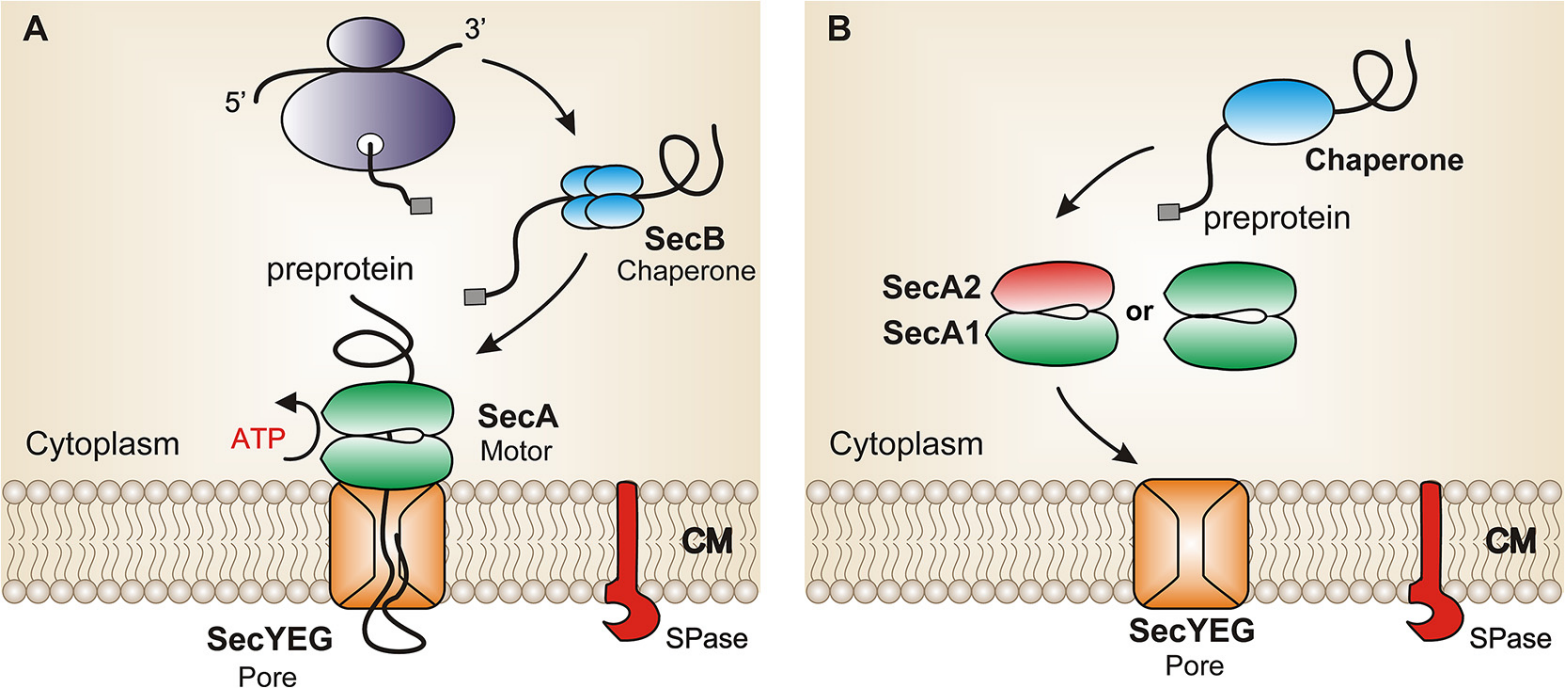
Model of protein secretion. A) The canonical secretion pathway, in which protein translocation process is mediated by protein-conducting pore SecYEG and the ATP-driven motor protein SecA. First, dimeric SecA binds to the SecYEG channel. The chaperone protein maintains the newly synthesized preproteins in their unfolded form and targets them to SecYEG bound SecA. SecA delivers the chemical energy by the cycles of ATP binding and hydrolysis to drive preproteins through SecYEG and across the cytoplasmic membrane. B) Proposed model of the mycobacterial SecA1/SecA2 pathway. SecA2 and SecA1 form a heterodimer and bind asymmetrically to the canonical SecYEG. The ATPase activity of either SecA2 or SecA1 or both then provides the translocation of the substrate through SecYEG and across the cytoplasmic membrane. SecA1 dimers or possibly SecA2 dimers may also work on their own. CM, cytoplasmic membrane. SPase, signal peptidase.

In bacteria, the majority of proteins are secreted across the cytoplasmic membrane via the general Sec-Pathway [1]. Protein transport in this pathway is mediated by the Sec translocase which consist of the protein-conducting pore SecYEG and the ATP-driven motor protein SecA [1–3] (Fig 1A). SecA delivers the chemical energy to drive proteins through the SecYEG pore in a post-translational manner [4–7]. In proteobacteria, the chaperone protein SecB plays an important role in maintaining the precursor protein in its unfolded form while targeting it to SecA [8,9].

Most bacteria have only a single *secA* gene. Both the structure and functional oligomeric state of this canonical SecA has been investigated with different methods and techniques. A multitude of studies demonstrate that SecA functions as a dimer during protein translocation [7,10–13]. It crystallizes as an antiparallel dimer [14–18] or a parallel dimer [19] and the exact arrangement of the functional dimer is unknown. The SecA dimer–monomer equilibrium is influenced by ligands and different conditions. SecA monomerizes in the presence of phospholipids [20,21] or synthetic signal peptides [22], although it also has been shown that SecA dimerizes with signal peptides [20]. In another study, SecA formed a dimer when bound to lipids and monomerized with the addition of nucleotides [23]. The apparently sensitive monomer-dimer equilibrium of SecA is also shifted towards the monomer at high salt concentrations and low temperature [24–26].

Previous studies have shown that some Gram-positive bacteria and mycobacteria have two different SecA proteins that are highly homologous to the *Escherichia coli* SecA, termed SecA1 and SecA2 [27–29]. A crystal structure of SecA1 has been obtained [16], but not for SecA2. Yet, sequence alignments and structural modeling predict that most functional domains are conserved between SecA1 and SecA2 [30,31]. SecA1 is essential and functions as the housekeeping SecA that associates with SecYEG to export the majority of secreted proteins [27]. SecA2 is not essential and seems to be especially important for the export of a subset of proteins which in some bacteria are virulence factors [32–35]. Previous studies with *Mycobacterium tuberculosis* SecA1 and SecA2 showed that both proteins function as ATPases and that the SecA2 ATPase activity is required for SecA2-mediated protein export [36–38]. SecA1 and SecA2 of *M*. *smegmatis* differ in subcellular localization; while SecA1 was found equally distributed between membrane and cytosolic fractions, SecA2 was predominantly cytosolic [37]. Recent studies in *M*. *tuberculosis* showed that SecA2 binds ADP with higher affinity than SecA1 [39].

In addition to SecA homologs, some Gram-positive bacteria also contain a homologue of SecY termed SecY2 [28,40]. In most of bacteria with SecA2 and SecY2 proteins, the genetic loci containing *sec* genes are highly conserved [40]. It has been suggested that the SecY2 protein combines with SecA2 and some accessory secretion proteins (ASPs) to form a separate translocase but SecY2 appears not essential for secretion [28,31]. Mycobacteria, however, do not contain an additional SecY protein and the genetic loci are not conserved [40]. Previous studies suggest that some sugar binding proteins in mycobacteria depend on both SecA1 and SecA2 for translocation [37] and it has been proposed that SecA2 might work together with the SecA1/SecYEG translocase [30]. Additionally, a study in *Streptococcus parasanguinis* showed that a large complex containing several *Streptococcus*-specific accessory proteins and SecA2 co-purifies with SecA1 [41], and recent studies in *Listeria monocytogenes* revealed that SecA2-dependent protein secretion requires SecA1 [42]. However, it is unclear whether SecA2 binds directly to SecA1 in order to export proteins through the SecYEG translocase. Furthermore, it is not known if SecA2 functions as a monomer or dimer in secretion of its specific substrates.

Here we characterized the mycobacterial SecA2 protein using biochemical and biophysical techniques. We used Microscale Thermophoresis (MST) and fluorescence cross-correlation spectroscopy (FCCS) to study the dimerization and interaction between SecA2 and SecA1.

## Materials and Methods

### Chemical and reagents

Cy5-maleimide was purchased from GE Healthcare and AlexaFluor 488-maleimide from Life Technologies. Cation exchange chromatography column HiTrap SP HP, Gel filtration chromatography column Superdex 200 10/300 GL, and NAP5 as well as Gel Filtration protein standards were purchased from GE Healthcare. Amicon ultracentrifugation filter was purchased from Merck Millipore and Ni-NTA Agarose was from Qiagen. PVDF membrane was purchased from Roche Diagnostics and anti-strep tag antibody was from IBA.

### Cloning of Mycobacterial SecA1 and SecA2

All strains and plasmids used are shown in Table 1. Plasmids for expression of SecA1 and SecA2 proteins were generated by PCR amplification of *secA1* and *secA2* gene from *M*. *tuberculosis strain* H37Rv genomic DNA (a gift from Prof. dr. Wilbert Bitter, University of Amsterdam). S*ecA1* PCR product was digested with NcoI and HindIII and cloned into pACYCDuet-1 yielding PIP151. *SecA2* PCR product was digested with NcoI and BamHI and cloned into pET15b yielding PIP152. An ATG start codon was added before the GTG codon for the expression in *E*. *coli*. For SecA2, the start codon used is a GTG located at nucleotide position 91 from the annotated start codon (NP_216337), since this represents the true start codon [36]. PIP151 and PIP152 were used as templates to generate other plasmids. A cysteine was introduced by site directed mutagenesis replacing serine at C-terminal of SecA1 (S945C) and SecA2 (S802C) yielding PIP153 and PIP154, respectively. His-tag sequences was added at C-terminal of SecA1 and Strep-tag sequences was added at C-terminal of SecA2 yielding PIP155 and PIP156, respectively. All cloning steps were verified by sequence analysis (Macrogen Europe).

**Table 1:** Strains and plasmids used in this study.

Strains/plasmids	Characteristics	Reference
DH5α	*supE44*, *ΔlacU169 (Δ80lacZ_M15) hsdR17*, *recA1*, *endA1*, *gyrA96 thi-1*, *relA1*	Previous study [43]
BL21(λDE3)	F—*omp*T *hsd*SB(rB–, mB–) *gal dcm* (λDE3)	Previous study [44]
PIP151	*SecA1* in pACYCDuet-1	This study
PIP152	*SecA2* in pET15b	This study
PIP153	SecA1(S945C) in pACYCDuet-1	This study
PIP154	SecA2(S802C) in pET15b	This study
PIP155	*SecA1* with C-terminal His-tag in pACYCDuet-1	This study
PIP156	*SecA2* with C-terminal Strep-tag in pET15b	This study

### Overexpression of Mycobacterial SecA1 and SecA2

*E*. *coli* BL21(λDE3) was transformed with the plasmids and used for expression and co-expression of SecA1 and SecA2. Cells were cultured at 30°C in LB supplemented with 34 μg/ml chloramphenicol for SecA1 expression or with 100 μg/ml ampicillin for SecA2 expression. For co-expression of SecA1 and SecA2, cells were cultured at 30°C in LB supplemented with 17 μg/ml chloramphenicol and 50 μg/ml ampicillin. Cells were grown to an optical density at 600 nm (OD_600_) of 0.6. Protein expression was induced by adding 1 mM isopropyl-D-thiogalactopyranoside (IPTG), and cells were grown further for 2 hours. Cells were harvested using the Beckman JLA 8.1000 rotor (7000 rpm, 15 min, 4°C), resuspended in 25 mM HEPES-KOH pH 6.5 and stored at -80°C. *E*. *coli* SecA was produced as described [45].

### Protein Purification and Labeling

Cells were lysed by French press treatment at 13000 psi and the lysates were centrifuged at 10.000 rpm for 15 min followed by ultracentrifugation at 100.000 rpm for 30 min. For purification, cell free extract was applied on a HiTrap SP HP column equilibrated with buffer A (20 mM HEPES-KOH, pH 6.5, 10% glycerol) as described [25]. The column was washed with buffer A supplemented with 100 mM NaCl and the protein was eluted with a linear 0.5 M NaCl gradient in buffer A. For labeling, SecA proteins were incubated with the fluorescent probe Cy5-maleimide or AlexaFluor 488 at pH 7.0 as described [25], and purified on Superose 12 gel filtration using buffer B (20 mM HEPES-KOH pH 7.5, 10% glycerol, 150 mM KCl). The labeling efficiency was determined by absorbance using a spectrophotometer. The extinction coefficients used were: ε_280_ = 90.650 cm^− 1^ M^− 1^ for *M*. *tuberculosis* SecA1, ε_280_ = 69.455 cm^− 1^ M^− 1^ for *M*. *tuberculosis* SecA2, ε_280_ = 76.000 cm^− 1^ M^− 1^ for *E*. *coli* SecA, ε_500_ = 72.000 cm^− 1^ M^− 1^ for AlexaFluor 488, and ε_649_ = 250.000 cm^− 1^ M^− 1^ for Cy5-maleimide. Labeling efficiencies were approximately 90% for *M*. *tuberculosis* SecA1 and SecA2, and 100% for *E*. *coli* SecA.

### Size Exclusion Chromatography

The protein was concentrated to obtain a concentration of 2–5 mg/ml using an Amicon ultracentrifugation filter. Size exclusion chromatography was carried out using a Superdex 200 10/300 GL column and eluted at 0.5 ml/min in buffer (20 mM HEPES-KOH, pH 7.5, 2 mM MgCl_2_, 50 mM arginine) supplemented with low (30 mM KCl) or high (300 mM KCl) salt. The peak protein fractions were detected and measured by the UV_280nm_ detector. Four protein standards; ovalbumin (43 kDa), conalbumin (75 kDa), aldolase (158 kDa) and ferritin (440 kDa), were used to estimate the molecular mass of SecA1 and SecA2.

### Microscale Thermophoresis

The microscale thermophoresis (MST) method has been described previously [46–48]. The dissociation constant (K_d_) of SecA1 and SecA2 dimers were measured using the Monolith NT.115 from Nanotemper Technologies. A solution of unlabeled protein was serially diluted and mixed with fluorescently labeled protein in high salt buffer (25 mM HEPES-KOH, pH 7.5, 300 mM KCl, 2 mM MgCl_2_, 50 mM arginine). The samples were incubated for 30 min on ice and then diluted in low salt buffer (25 mM HEPES-KOH, pH 7.5, 30 mM KCl, 2 mM MgCl_2_, 50 mM arginine). The end concentration of the unlabeled protein was varied from 1 nM to 5 μM and for fluorescently labeled protein, it was 25 nM. The samples were loaded into Monolit NT.115 capillaries after incubation at room temperature for 10 minutes. MST measurements were performed by using 80% LED power and 80% IR-laser power. Data analyses were performed using Nanotemper Analysis software.

### Fluorescence Cross-Correlation Spectroscopy

Fluorescence cross-correlation spectroscopy (FCCS) experiments were performed on a dual-color laser scanning LSM710 inverted confocal microscope (Zeiss GmbH). He-Ne laser at 488 nm and an argon laser at 633 nm were used to excite the fluorescently labeled proteins. The fluorescence was split into two channels of 505–610 nm (AlexaFluor 488 emission) and 655–710 nm (Cy5 emission) by a dichroic beam splitter. SecA1-Cy5 and SecA2-AF488 were mixed at 1:1 ratio and incubated in the presence of 300 mM KCl for 30 min on ice. The samples were loaded on NAP5 columns and eluted with 25 mM HEPES-KOH, pH 7.5, 30 mM KCl, 2 mM MgCl_2_, 50 mM arginine. The samples were incubated for 10 minutes in room temperature prior to FCCS measurements. FCCS measurement and data analysis were performed as described previously [49].

### Pulldown Assay

Cells expressing both His_6_-SecA1 and Strep-SecA2 were resuspended in 25 mM HEPES-KOH, pH 7.5, 300 mM KCl and lysed by French press treatment at 13000 psi. The cell lysates were diluted in 25 mM HEPES-KOH, pH 7.5, 100 mM KCl, incubated for 30 min on ice, and applied to Ni-NTA agarose. The samples were washed with 25 mM HEPES-KOH, pH 7.5, 100 mM KCl and they were eluted with 25 mM HEPES, pH 7.5, 100 mM KCl, 300 mM imidazole. The proteins were analyzed by SDS-PAGE and visualized by Coomassie blue staining. The proteins were also transferred to a PVDF membrane for Western blot analysis with anti-strep tag antibody. An experiment with cell lysates of His_6_-SecA1 only, Strep-SecA2 only, and an empty vector were also carried out and analyzed as a control.

## Results

### Mycobacterial SecA1 and SecA2 individually form homodimers

**Fig 2:**
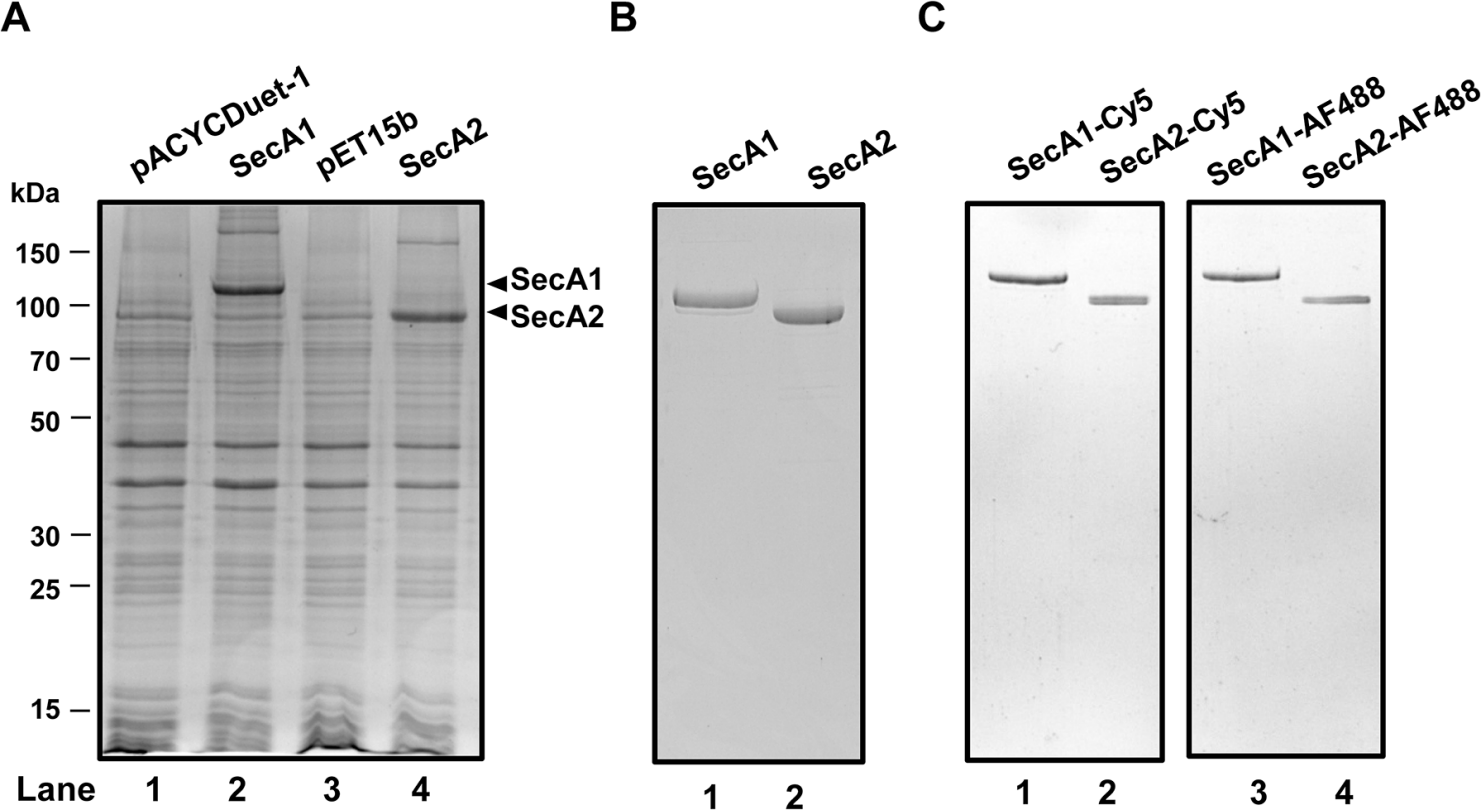
Overexpression, purification, and fluorescent labeling of *M*. *tuberculosis* SecA1 and SecA2. A) Overexpression of *M*. *tuberculosis* SecA1 (lane 2) and SecA2 (lane 4) in *E*. *coli* BL21(λDE3) with a molecular mass of 105 and 85 kDa, respectively. Molecular masses of protein standard are indicated on the left. The empty vector, pACYCDuet-1 and pET15b, are shown as controls (lane 1 and 3). B) Coomassie-stained SDS-PAGE of purified *M*. *tuberculosis* SecA1 (lane 1) and SecA2 (lane 2). C) Visualization of fluorescently labeled SecA1 and SecA2 in SDS-PAGE by fluorescence imaging. SecA1 and SecA2 were labeled with the fluorescent probe Cy5 (lane 1 and 2) or AF488 (lane 3 and 4).

**Fig 3:**
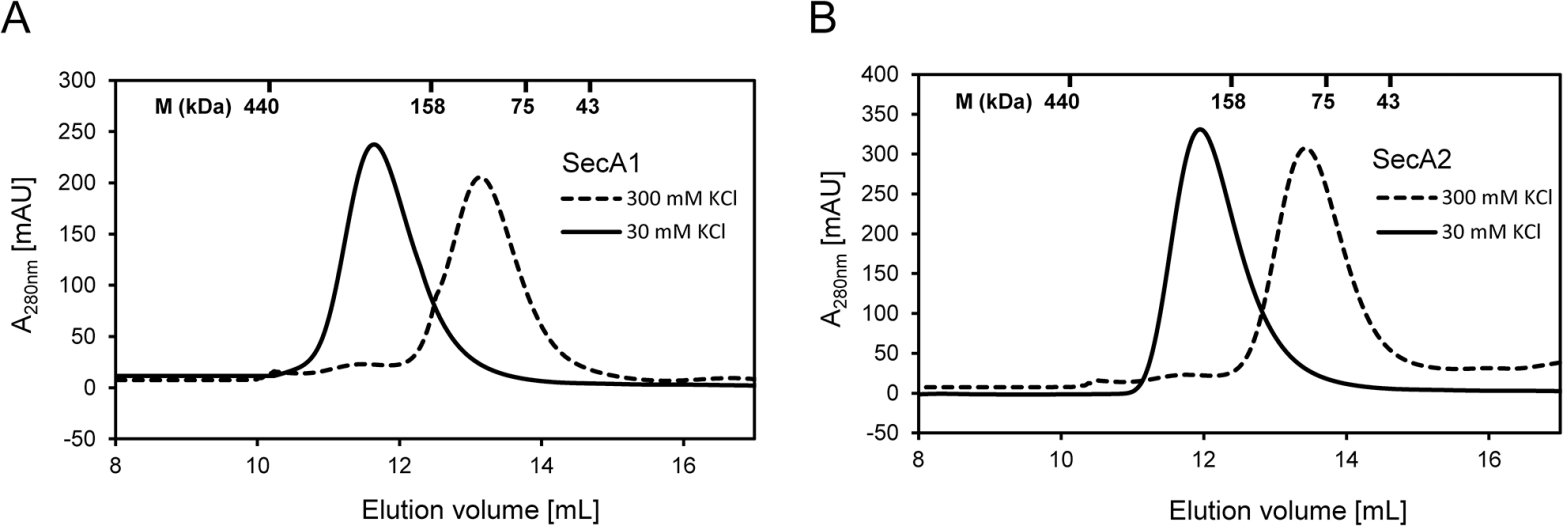
Size Exclusion Chromatography on SecA1 and SecA2. The experiments were carried out in low salt buffer (30 mM KCl) and high salt buffer (300 mM KCl). The signals from the UV_280nm_ detector are shown. The molecular masses (M) and the elution volumes of the peaks of the calibration standards are indicated along the top X axis. A) SEC elution profile of SecA1 in high salt and low salt buffer. The peak eluted at ∼13.5 ml and ∼11.5 ml contain monomeric and dimeric SecA1, respectively. B) SEC elution profile of SecA2 in high salt and low salt buffer. The peak eluted at ∼14 ml and ∼12 ml contain monomeric and dimeric SecA2, respectively.

Previous studies on the canonical *E*. *coli* SecA show that it exists as a dimer in the cytosol and when bound to SecYEG [7,25,26,50,51]. Here we examined the oligomeric state of the mycobacterial SecA homologues, SecA1 and SecA2, using biochemical and biophysical techniques. The SecA1 and SecA2 proteins were expressed in *E*. *coli* (Fig 2A) and purified using cation exchange chromatography (Fig 2A and S1 Fig). The purified SecA1 and SecA2 were devoid from endogenous *E*. *coli* SecA as demonstrated by immunoblotting (S1 Fig). The purified proteins were then subjected to size exclusion chromatography (SEC). SEC experiments were done in buffer containing low (30 mM KCl) or high salt (300 mM KCl), since the presence of high salt has been shown to affect the dimerization of *E*. *coli* SecA [24–26]. We used protein standards to estimate the molecular mass of SecA1 and SecA2. The SEC elution profile of SecA1 shows a peak eluting at ∼11.5 mL in low salt buffer, presumably containing dimeric SecA1, and a peak at ∼13.5 ml in high salt buffer indicating monomerization of SecA1 (Fig 3A). In case of SecA2, the peak elutes at ∼12 mL in low salt buffer and at ∼14 ml in high salt buffer likely to contain dimeric and monomeric SecA2, respectively (Fig 3B) confirming a similar SEC result that has been shown previously for SecA2 [39].

**Fig 4:**
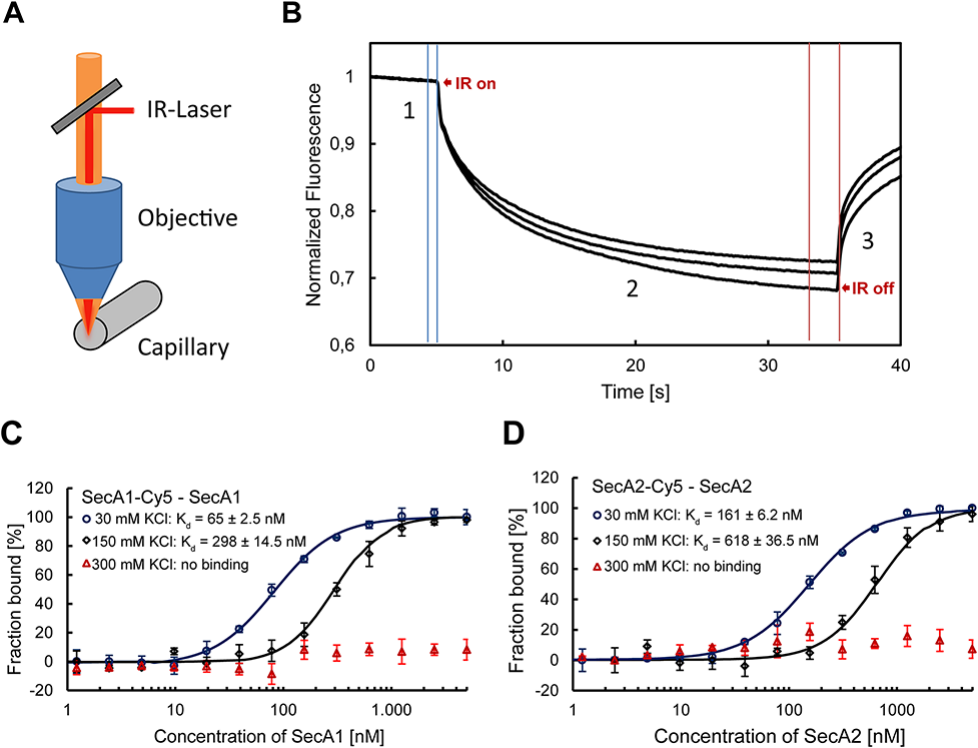
MST analysis of SecA1 and SecA2 homodimerization. A) Scheme of the MST experiments and B) diffusion traces observed in MST. The mobility of molecules in a temperature gradient is followed by the fluorescence intensity in a central spot. When the IR-Laser is turned on, the initial fluorescence (1) drops due to the thermophoretic movement of fluorescently labeled proteins out of the heated spot (2). When the IR-Laser is turned off, back-diffusion of the fluorescently labeled proteins is observed which is driven by mass diffusion and depends on the hydration shell of the proteins (3). Dimers diffuse slower than monomers. SecA1 (C) and SecA2 (D) dimerization measured by MST. Unlabeled protein (1 nM to 10 μM) was titrated into a fixed concentration of labeled protein (25 nM). The thermophoretic signal is plotted as a function of the protein concentration resulting in a dimerization curve. The curves were fitted using the Hill-equation and apparent K_d_ values were determined. Error bars represent the standard error of 3 measurements. The apparent K_d_ for SecA1 and SecA2 dimerization at low salt concentrations were 65 ± 2.5 nM and 161 ± 6.2 nM, respectively. The measurement of samples at high salt concentrations showed no binding.

In order to quantitatively determine the binding affinity of the SecA1 and SecA2 homodimers, we used Microscale Thermophoresis (MST) technique. MST enables the quantitative analysis of protein interactions in solution at picomolar concentrations. This technique measures the motion of molecules in temperature gradients, and detects changes in their molecular properties, such as size, conformation, charge, and hydration shell [52]. An infrared laser is used for local heating of the sample inside a capillary, and the mobility of molecules in a temperature gradient is followed by fluorescence (Fig 4A and 4B). The binding parameters are obtained by titrating the fluorescently labeled molecule with increasing concentrations of the binding partner. This technique has been used previously to determine the binding affinity of protein-protein interactions in different buffer condition including high salt and low salt concentrations [46–48]. To detect the thermophoretic movement, SecA1 and SecA2 proteins were labeled with the fluorescent probe Cy5 (Fig 2C). To determine the protein binding, fluorescently labeled SecA1 or SecA2 at a constant concentration was added to increasing concentrations of unlabeled SecA1 or SecA2 in buffer containing high salt (300 mM KCl). In this condition we assumed that most of the proteins are in their monomeric form. To again allow dimerization, the samples were diluted with buffer containing low salt (30 mM KCl) and incubated prior to the MST measurement. With increasing protein concentration the monomer-dimer equilibrium shifts towards the dimer, which results in a different mobility in the temperature gradient due to the change of their hydration shell. Strong binding of SecA1-SecA1 was observed with an apparent dimer dissociation constant (K_d_) of 65 ± 2.5 nM at 30 mM KCl. The experiment of the samples at moderate salt concentrations (150 mM KCl) showed the lower binding affinity with a K_d_ of 298 ± 14.5 nM, and no binding was observed at 300 mM KCl (Fig 4C). These results show that increased ionic strength will shift the K_d_ for SecA1 dimerization to higher protein concentrations. At high salt concentration, SecA1 proteins exist in the monomeric form, while at low salt concentration they form homodimers. In case of SecA2, the dimerization occurred with a K_d_ of 161 ± 6.2 nM at 30 mM KCl, and a K_d_ of 618 ± 36.5 nM at 150 mM KCl, while at 300 mM KCl no binding was observed (Fig 4D), demonstrating that also the SecA2 dimer is salt-sensitive. In good agreement with SEC data, these results show that both mycobacterial SecA1 and SecA2 proteins form dimer at low salt concentration and dissociate to monomers at high salt concentration.

### SecA1 interact with SecA2 to form heterodimers

**Fig 5:**
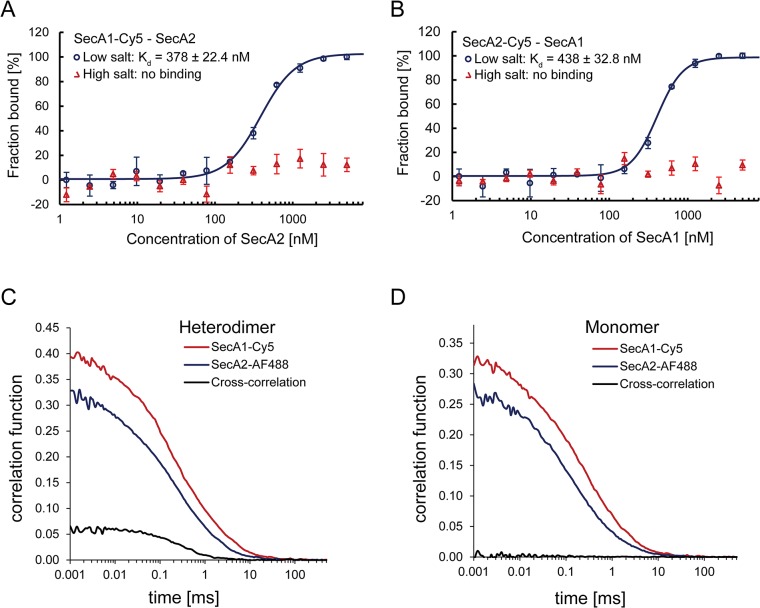
Interactions of SecA1 and SecA2. A) MST measurement of SecA1-Cy5 titrated with increasing concentrations of unlabeled SecA2 B) MST measurement of SecA2-Cy5 titrated with increasing concentrations of unlabeled SecA1. The curves were fitted using the Hill-equation and the apparent K_d_s were determined. Error bars represent the standard error of 3 measurements. The formation of the SecA1/SecA2 heterodimer is observed with an apparent K_d_ of 378 ± 22.4 nM and 438 ± 32.8 nM. C) FCCS analysis on SecA1 and SecA2. SecA1-Cy5 and SecA2-AF488 were mixed at ratio of 1:1. The measurements were done in 10 s and 10 repetitions. Auto-correlations of SecA1-Cy5 (red curve), SecA2-AF488 (blue curve) and cross-correlations (black curve) were recorded. The amplitude of cross-correlation was compared to the amplitude of autocorrelation. Positive cross-correlations is observed indicating the heterodimerization of SecA1 and SecA2. D) A control experiment of samples at high salt concentrations showed no cross-correlations indicating that the proteins stay in monomeric form.

Next, we investigated the interaction between the SecA1 and SecA2 proteins with the MST technique. SecA1 was labeled with the fluorescent probe Cy5 as described before. Increasing concentrations of unlabeled SecA2 in high salt buffer were added to SecA1-Cy5 at a constant concentration. The samples were diluted with low salt buffer and subjected to MST measurements. As shown in Fig 5A, heterodimerization of SecA1 and SecA2 was observed with an apparent K_d_ of 378 ± 22.4 nM while the experiment in high salt buffer showed no interaction. To confirm the heterodimerization we performed a MST experiment with SecA2-Cy5 titrated with increasing concentrations of unlabeled SecA1. A similar affinity with an apparent K_d_ of 438 ± 32.8 nM was observed confirming heterodimerization of SecA1 with SecA2 (Fig 5B). The estimated K_d_ values indicate that SecA1/SecA2 heterodimer has a significantly lower affinity than the SecA1 or SecA2 homodimer. To confirm the specificity of the SecA1 and SecA2 interaction, control experiments were conducted with *E*. *coli* SecA. No interaction was observed between *E*. *coli* SecA and the *M*. *tuberculosis* SecA1 or SecA2 protein (S2 Fig), whereas homodimerization of the *E*. *coli* SecA could be detected with an apparent K_d_ of 31 ± 1.5 nM. These data demonstrate that SecA1-SecA2 dimerization observed with the MST is specific.

In order to compare our data from the MST measurements with an independent method, we also measured the SecA1 and SecA2 interaction using a dual-color confocal fluorescent microscope and fluorescence cross-correlation spectroscopy (FCCS). FCCS allows the characterization of heterodimers whose subunits are labeled with two spectrally distinct fluorophores [53]. This technique detects fluorescent fluctuations when fluorescently labeled proteins diffuse through the two spatially aligned laser foci of a confocal microscope that excite the fluorophores. If the two subunits of dual-labeled heterodimers co-migrate through the laser foci, both fluorophores are excited at the same time and a co-fluctuation is recorded. Since FCCS also detects singly labeled proteins at the same time, it allows one to quantitatively determine the fraction of dual-color heterodimer. For the FCCS experiment, SecA1 was labeled with Cy5 and SecA2 was labeled with AlexaFluor 488 (Fig 2C). Approximately similar concentrations of SecA1-Cy5 and SecA2-AF488 were mixed and incubated at high salt concentration to allow for monomerization. Dimerization was obtained by buffer exchange to low salt concentration using gel filtration. The samples were then incubated for 10 minutes at room temperature, measured and analyzed with FCCS. The concentration of the dual-color heterodimers can be calculated from the amplitude of the cross-correlation function and related to the total concentration of fluorescently labeled proteins, which is derived from the auto-correlation curves of the individual colors (SecA1-Cy5 or SecA2-AF488). A positive cross-correlation signal was observed for SecA1-Cy5 and SecA2-AF488 at low salt concentrations, showing that 12% of all proteins co-migrated as heterodimers (Fig 5C). No cross-correlations was observed for the samples at high salt concentration, indicating that in this condition the proteins exist in monomeric form (Fig 5D). The 12% co-migration is an underestimate of the actual fraction of heterodimers, as the mixing experiment stochastically should result in the formation of homo- and heterodimers. However, since the yield also depends on the relative affinities of interaction that differ for homo- and heterdimerization, an exact estimate of the extent of heterodimer formation cannot be made.

**Fig 6:**
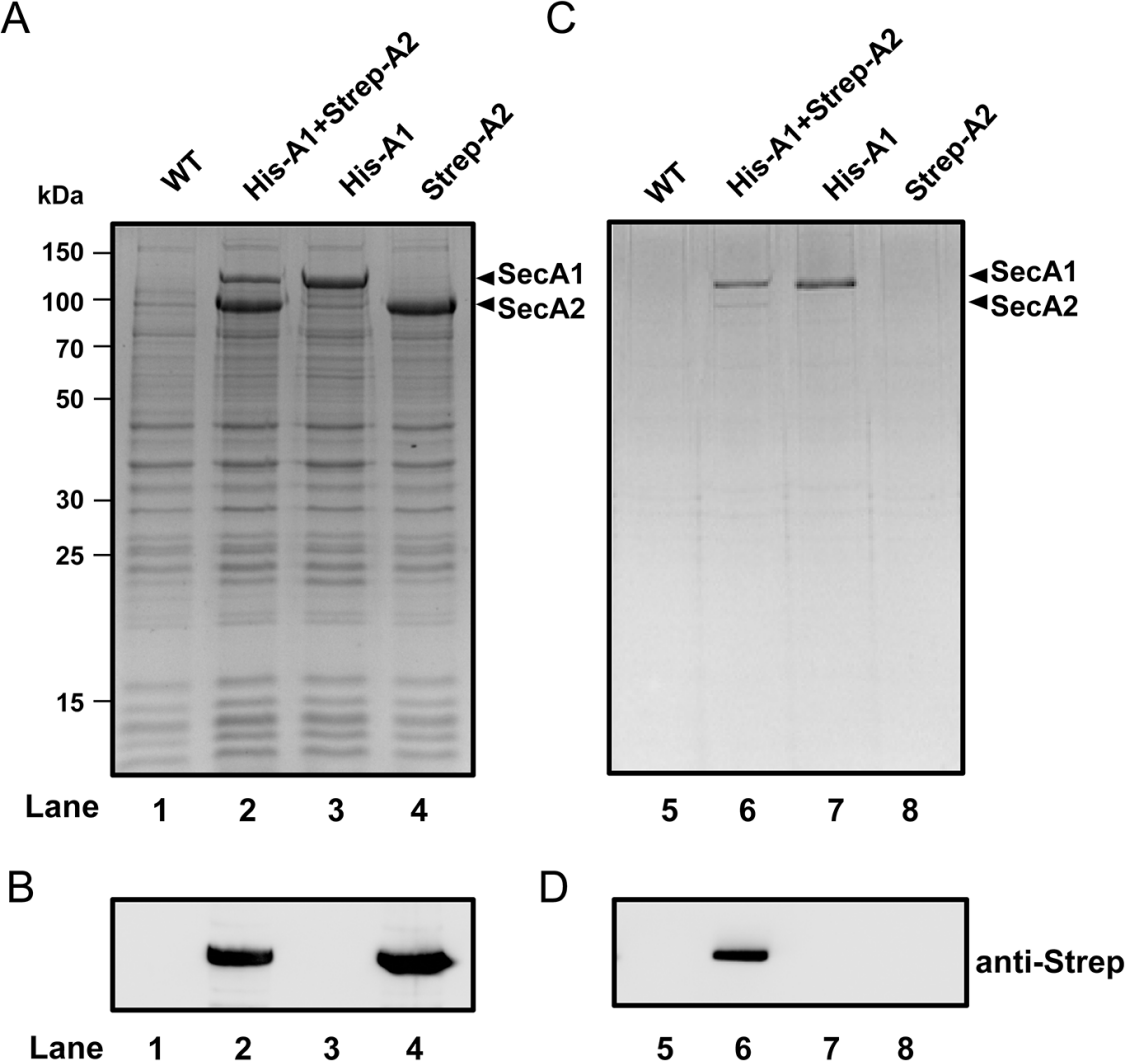
Pulldown analysis on SecA1 and SecA2. A) Coomassie-stained SDS-PAGE of cell lysate of wild type (lane1), co-expressed His_6_-SecA1 and Strep-SecA2 (lane 2), His_6_-SecA1 only (lane 3), and Strep-SecA2 only (lane 4). Molecular masses of protein standard are indicated on the left. B) Western blot analysis of cell lysate of wild type (lane1), co-expressed His_6_-SecA1 and Strep-SecA2 (lane 2), His_6_-SecA1 only (lane 3), and Strep-SecA2 only (lane 4) against α-Strep antibody. C) Coomassie-stained SDS-PAGE of purified His_6_-SecA1 (lane 5–8). D) Western blot analysis of purified His_6_-SecA1 against α-Strep antibody (lane 5–8). Western blot data showed that SecA2 was co-purified with SecA1 (lane 6).

To confirm SecA1 and SecA2 association with a classical biochemical technique, we performed pulldown experiments at physiological ionic conditions. For purification and immunodetection, a hexa-histidine tag and strep-tag were conjugated to the C-terminus of SecA1 and SecA2, respectively. His_6_-SecA1 and Strep-SecA2 were co-expressed in *E*. *coli* and were co-purified with Ni-NTA agarose. All the protein fractions were analyzed with SDS-PAGE and western blot using anti-strep tag antibodies. We found that strep-tagged SecA2 co-purified with his-tagged SecA1 on Ni-NTA affinity chromatography (Fig 6D). Our result from pulldown experiment is in good agreement with MST and FCCS data that demonstrate that mycobacterial SecA2 indeed can associate with SecA1 to form a heterodimer.

## Discussion

The accessory SecA2 system in bacteria has been studied extensively during the past decade [30,31]. It has been proposed that SecA2 might cooperate with SecA1 in order to export its protein substrates through the SecYEG translocon. However, if and how SecA1 and SecA2 interact is still poorly understood. It was also unknown if SecA2 functions as a monomer or dimer to export the specific preprotein. Investigating the interaction between SecA2 and SecA1/SecYEG translocase might give a better understanding on SecA2 function in protein translocation.

In this paper, we examined the oligomeric state of SecA2 and its physical interactions with SecA1 in solution. First, we determined the dimerization of individual SecA1 and SecA2 by two independent techniques, SEC and MST. The experiments were done in the presence of both high and low ionic strength, since the electrostatic interaction has been shown to play a critical role in dimerization of the *E*. *coli* SecA [24–26]. Our data from SEC showed that both SecA1 and SecA2 are predominantly monomeric at high salt and dimeric at low salt concentration, similar to *E*. *coli* SecA (Fig 3A and 3B). From the MST experiments, we found that SecA1 forms homodimers with an apparent K_d_ of 65 ± 2.5 nM (Fig 4C). This dimer dissociation constant is higher than described in previous studies for *E*. *coli* SecA (0.8–14 nM) [24,25] but still indicates a high affinity interaction. Similar to SecA1, SecA2 was found to form homodimers with an apparent K_d_ of 161 ± 6.2 nM, which is about 2.5-fold lower than the K_d_ of SecA1 homodimerization (Fig 4D). The experiments at high salt concentration show no binding of SecA1 or SecA2 confirming that electrostatic interactions are as important for dimerization as in the *E*. *coli* SecA dimer [24–26] consistent also with the salt-dependent SecA1 or SecA2 homodimer formation seen with SEC (Fig 3A and 3B). In comparison with the K_d_ of the *E*. *coli* SecA dimer, the lower affinity of the mycobacterial SecA1 and SecA2 homodimers may facilitate the formation of SecA1/2 heterodimers under conditions in the cell that require secretion of SecA2 specific substrates. The concentration of potassium ions in the cytoplasm of cells grown in low-osmolarity growth medium is approximately 140 mM [54]. Since the cellular concentration of SecA is estimated to be in micromolar range (5–8 μM) [50,55], our K_d_ data indicate that most of SecA1 and SecA2 are dimeric, likely as homodimers in the cell. However, in high-osmolarity growth medium, the cytoplasmic K^+^ concentration increases to approximately 760 mM [54], which may affect the dimerization of SecA1 and SecA2.

Our data also show the interaction of SecA1 and SecA2 in solution, likely representing heterodimerization. The MST data demonstrate that SecA1 interacts with SecA2 with significantly higher dissociation constants than the homodimer (K_d_ of 378 ± 22.4 nM for SecA1/SecA2 and K_d_ of 65 ± 2.5 nM for the SecA1 homodimer). Thus, the K_d_ value of the heterodimer is about 6-fold lower than the K_d_ of a SecA1 homodimer and 3-fold lower than the K_d_ of a SecA2 homodimer. SecA2 could also directly interact with SecYEG and drive translocation as it has ATPase activity [36]. However, in comparison to the canonical SecA, the mycobacterial SecA2 has a deletion in the C-terminal domain (CTD) and the helical wing domain (HWD) [30,31]. In *E*. *coli* SecA, CTD is important for interaction with phospholipid [56], while HWD has been suggested to be important for the interaction with SecYEG [57,58]. Additionally, the notion that SecA2 is predominantly cytosolic, while SecA1 is equally distributed between membrane and cytosol [37] suggesting that SecA2 may not bind directly to SecYEG in the membrane. Although SecA2 and SecYEG association has been proposed based on a genetic study in *M*. *smegmatis* [59], there is no evidence that SecA2 directly interacts with SecYEG. Our observations on the efficient SecA1 and SecA2 interaction suggests that the SecA1/SecA2 heterodimer may functional interact with SecYEG in *M*. *tuberculosis*. Furthermore, an asymmetric arrangement of the *E*. *coli* SecA dimer bound to SecYEG that retains the sensitivity of the dimerization interface suggests that also in the SecA1/SecA2 heterodimer only one subunit, possibly SecA1 may be bound to SecYEG while SecA2 may interact with the SecA1-SecYEG complex via dimerization [13,25].

We confirm the interaction between SecA1 and SecA2 by two further independent approaches, i.e., biochemical pulldown and FCCS experiments with fluorescently labeled SecA1 and SecA2. The pulldown analysis showed the heterodimerization of SecA1 and SecA2 at physiological ionic conditions. FCCS data suggest that in solution the amount of homodimeric form of SecA1 or SecA2 is higher than the heterodimeric one as only 12% of proteins were found in heterodimers whereas in a entirely stochastically process, this may amount to maximal 50%.

In summary, we demonstrate a physical interaction between mycobacterial SecA1 and SecA2 proteins. Since some secretory proteins require both SecA1 and SecA2, our data lead to the suggestion that a SecA1/SecA2 heterodimer functions in the export of SecA2-specific proteins through the SecYEG translocase. However, the biological function of the homo- and heterodimers and how they interact with SecYEG still needs to be investigated. Similarly to the asymmetric association of the canonical SecA-dimer with SecYEG [13,25] where one SecA protomer binds tightly to SecYEG, whereas the second SecA protomer binds to the SecYEG-bound SecA protomer (Fig 1A) we propose that SecA1/SecA2 heterodimer may also asymmetrically interact with SecYEG (Fig 1B).

## Supporting Information

S1 Fig SDS-PAGE and western blot analysis of purified *M*. *tuberculosis* SecA1 and SecA2. A) Coomassie-stained SDS-PAGE of purified *M*. *tuberculosis* SecA1 (lane 1), SecA2 (lane 2), and *E*. *coli* SecA (lane 3) and B) western blot using an anti-*E*. *coli* SecA antibody.
(DOCX)

S2 Fig MST analysis on the interaction between *E*. *coli* SecA and *M*. *tuberculosis* SecA1 and SecA2. MST measurement of *E*. *coli* SecA-Cy5 (open circles), *M*. *tuberculosis* SecA1-Cy5 (open triangles), and SecA2-Cy5 (open square) titrated with increasing concentrations of unlabeled *E*. *coli* SecA, The curves were fitted using the Hill-equation and the apparent K_d_ values were determined with a standard error of 3 measurements. The formation of the SecA homodimer showed an apparent K_d_ of 31 ± 1.5 nM, whereas for the *M*. *tuberculosis* SecA proteins, no interaction with *E*. *coli* SecA was observed.
(DOCX)
