# Supplementary material for: In Vitro Interaction of the Housekeeping SecA1 with the Accessory SecA2 Protein of Mycobacterium tuberculosis
Source: PLoS One. 2015 Jun 5;10(6):e0128788. doi: 10.1371/journal.pone.0128788 (PMC4457860; doi:10.1371/journal.pone.0128788)
Supplement: S1 Fig — A) Coomassie-stained SDS-PAGE of purified M. tuberculosis SecA1 (lane 1), SecA2 (lane 2), and E. coli SecA (lane 3) and B) western blot using an anti-E. coli SecA antibody. (DOCX) [file pone.0128788.s001.docx]

**
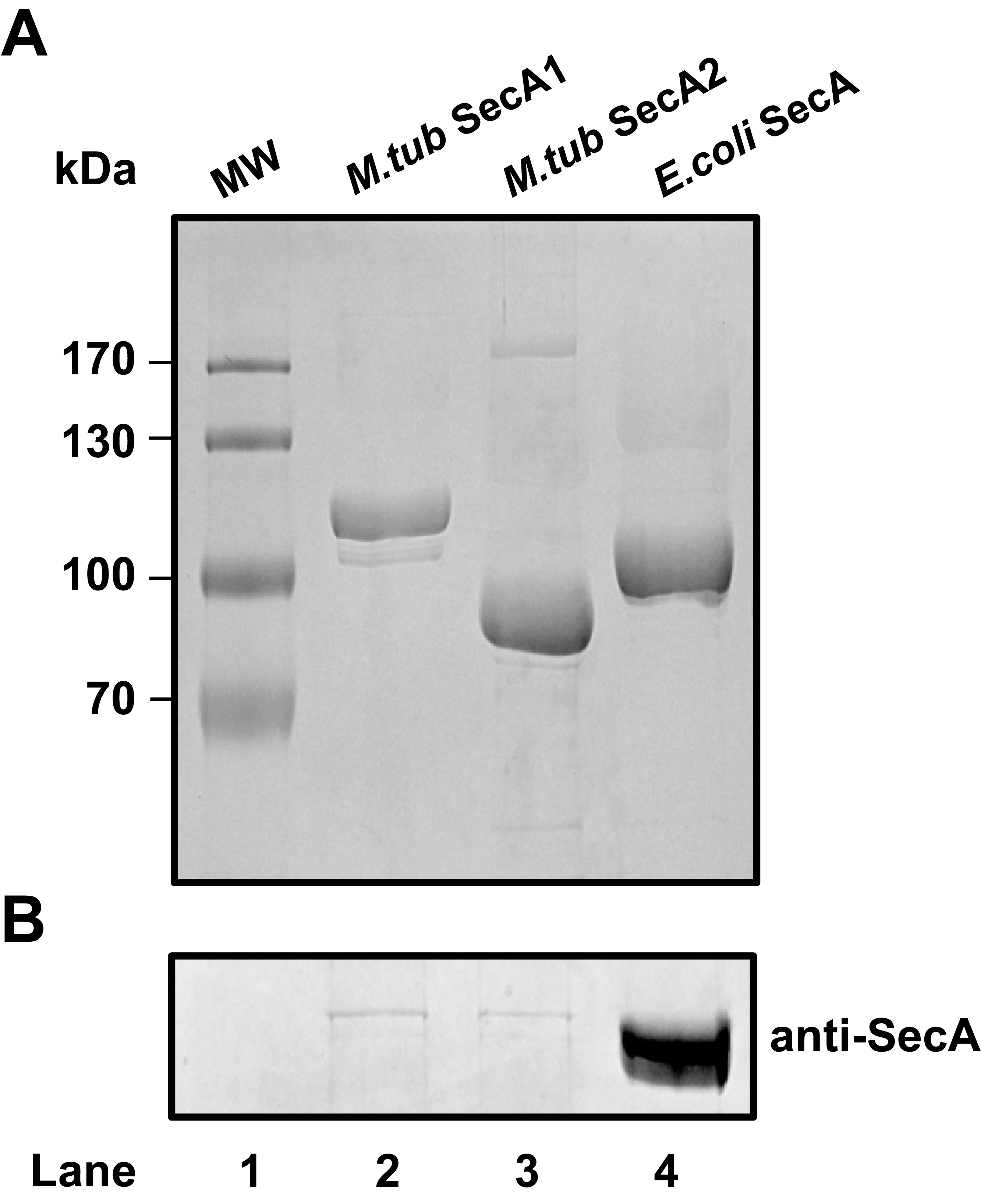
**

**S1 Fig. SDS-PAGE and western blot analysis of purified *M. tuberculosis* SecA1 and SecA2.** A) Coomassie-stained SDS-PAGE of purified *M. tuberculosis* SecA1 (lane 1), SecA2 (lane 2), and *E. coli* SecA (lane 3) and B) western blot using an anti-*E. coli* SecA antibody.
