# Supplementary material for: In Vitro Interaction of the Housekeeping SecA1 with the Accessory SecA2 Protein of Mycobacterium tuberculosis
Source: PLoS One. 2015 Jun 5;10(6):e0128788. doi: 10.1371/journal.pone.0128788 (PMC4457860; doi:10.1371/journal.pone.0128788)
Supplement: S2 Fig — MST measurement of E. coli SecA-Cy5 (open circles), M. tuberculosis SecA1-Cy5 (open triangles), and SecA2-Cy5 (open square) titrated with increasing concentrations of unlabeled E. coli SecA, The curves were fitted using the Hill-equation and the apparent Kd values were determined with a standard error of 3 measurements. The formation of the SecA homodimer showed an apparent Kd of 31 ± 1.5 nM, whereas for the M. tuberculosis SecA proteins, no interaction with E. coli SecA was observed. (DOCX) [file pone.0128788.s002.docx]

**
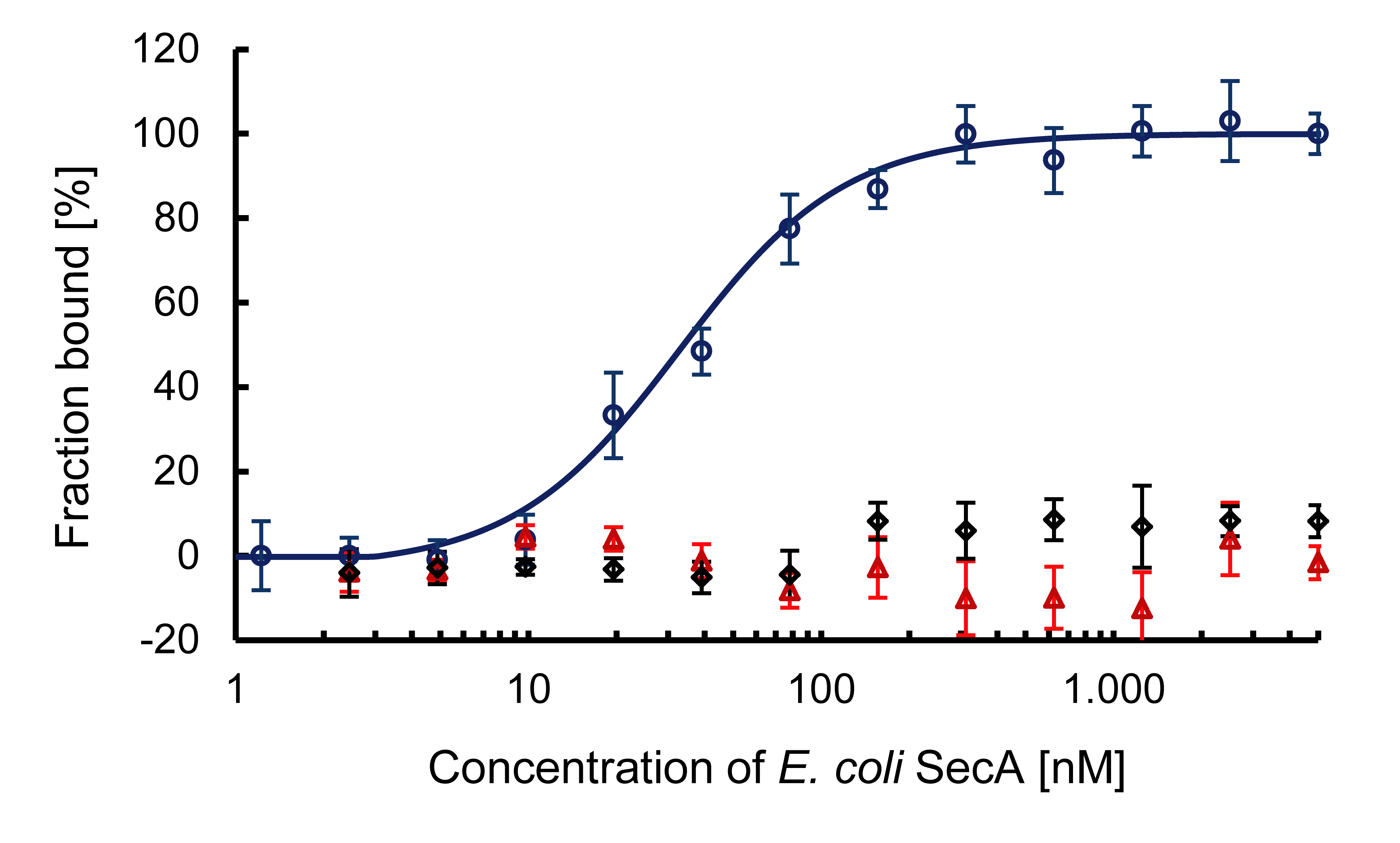
S2 Fig. MST analysis on the interaction between *E. coli* SecA and *M. tuberculosis* SecA1 and SecA2.** MST measurement of *E. coli* SecA-Cy5 (open circles), *M. tuberculosis* SecA1-Cy5 (open triangles), and SecA2-Cy5 (open square) titrated with increasing concentrations of unlabeled *E. coli* SecA, The curves were fitted using the Hill-equation and the apparent K_d_ values were determined with a standard error of 3 measurements. The formation of the SecA homodimer showed an apparent K_d_ of 31 ± 1.5 nM, whereas for the *M. tuberculosis* SecA proteins, no interaction with *E. coli* SecA was observed.
